# Pulmonary Cavitary Sarcoidosis: A Case Report

**DOI:** 10.7759/cureus.46398

**Published:** 2023-10-03

**Authors:** Bhanu Shrestha, Ravi Mahat

**Affiliations:** 1 Department of Respiratory Medicine, Karuna Hospital, Kathmandu, NPL; 2 Department of Respiratory Medicine, Grande International Hospital, Kathmandu, NPL

**Keywords:** flexible bronchoscopy, hilar lymphadenopathy, cavitary lung lesion, active pulmonary tuberculosis, pulmonary sarcoidosis

## Abstract

Sarcoidosis is a granulomatous disease characterized by a non-caseating granuloma formation in different organs of the body. However, the presence of cavitary lesions is rare. We present a case report of a 38-year-old male who presented with a three-month history of cough, dyspnea, and weight loss. Computed tomography of the chest demonstrated enlarged mediastinal and bilateral hilar lymphadenopathy with bilateral perihilar consolidation and cavitation in the upper lobes of both lungs. Later, the patient underwent bronchoscopy with bronchoalveolar lavage and endobronchial biopsy which showed well-formed and non-necrotizing granulomas which were also embedded in the dense hyaline sclerosis. This finding is consistent with sarcoidosis. Treatment with systemic corticosteroids was initiated, resulting in significant improvement in the patient’s symptoms. This case report highlights the uncommon manifestation of pulmonary cavitary sarcoidosis and emphasizes the significance of accurate diagnosis and appropriate management of this complex disease.

## Introduction

Sarcoidosis is a multisystem granulomatous disease, most commonly involving the respiratory system [1]. However, cavitation as the presenting manifestation, called primary cavitary sarcoidosis (PCS), is rare and observed in fewer than 0.5% of people suffering from sarcoidosis [2]. The differential diagnosis for cavitary lung lesions is broad, including tuberculosis, various infections, vasculitis, etc. [3]. To contribute to the understanding, we present a case report describing the clinical features, diagnosis, and treatment of a patient with PCS [4]. This report underscores the importance of a comprehensive approach involving various diagnostic modalities and the need for considering sarcoidosis as a differential diagnosis of cavitary lung lesions for appropriate management.

## Case presentation

A 38-year-old male, a security personnel, presented with mild cough and dyspnea, which increased when hurrying on ground level or walking up a slight hill, and weight loss for three months. His past medical history was significant for epistaxis one year back due to a decrease in platelet count for which a single pint of platelet concentrate was transfused. His thrombocytopenia improved with treatment. He denied any history of tuberculosis or contact with tuberculosis patients.

His vital signs were normal except for a lower blood oxygen saturation level, which was 93% on room air. He had clubbing and inspiratory crepitation on the left axillary area. He had no discernible skin lesions, eye symptoms, or joint pain.

Angiotensin-converting enzyme (ACE) level was 139 U/L (normal range = 12-68 U/L). Antinuclear antibodies, rheumatoid factor, Mantoux test, and antineutrophil cytoplasmic antibodies were negative. Similarly, other investigations such as 24-hour urinary calcium excretion, complete blood count, liver function test, renal function test, calcium level, and vitamin D level were within normal limits.

**Figure 1:**
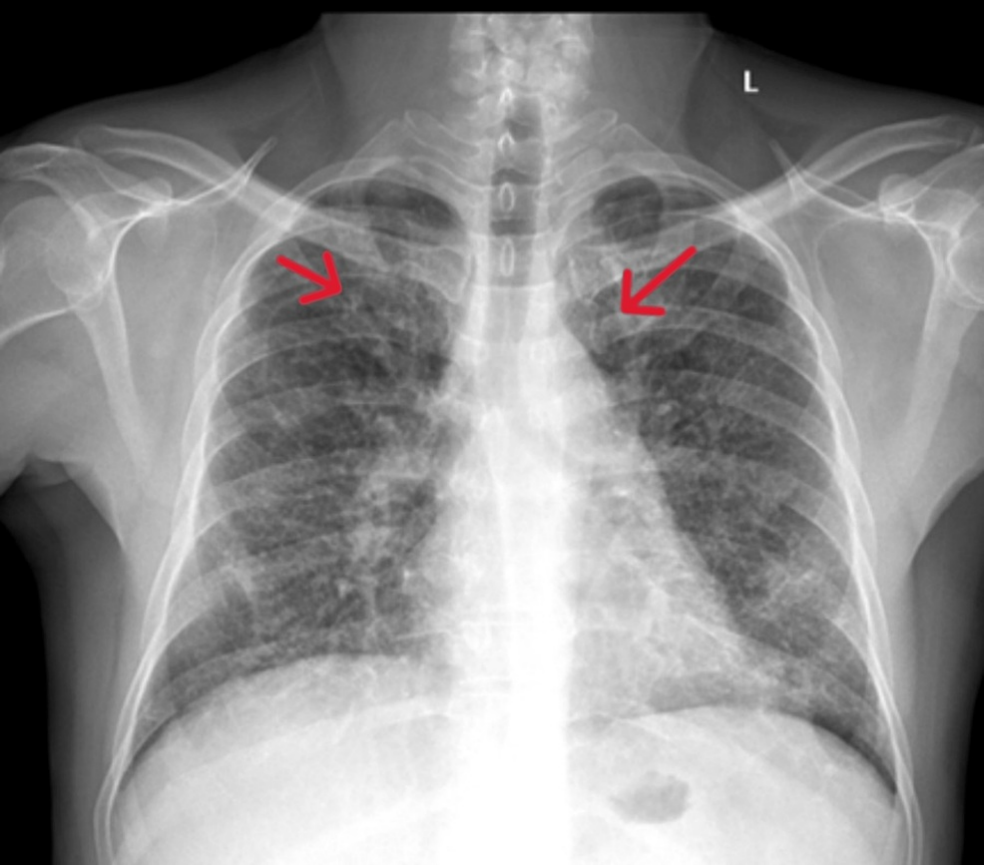
Chest X-ray showing bilateral pulmonary opacities on both upper lobes.

Spirometry demonstrated a mild restrictive pattern. Similarly, ultrasonography of the abdomen and pelvis showed splenomegaly and periportal lymphadenopathy. Chest X-ray (CXR) revealed bilateral pulmonary opacities on both upper lobes (Figure 1).

**Figure 2:**
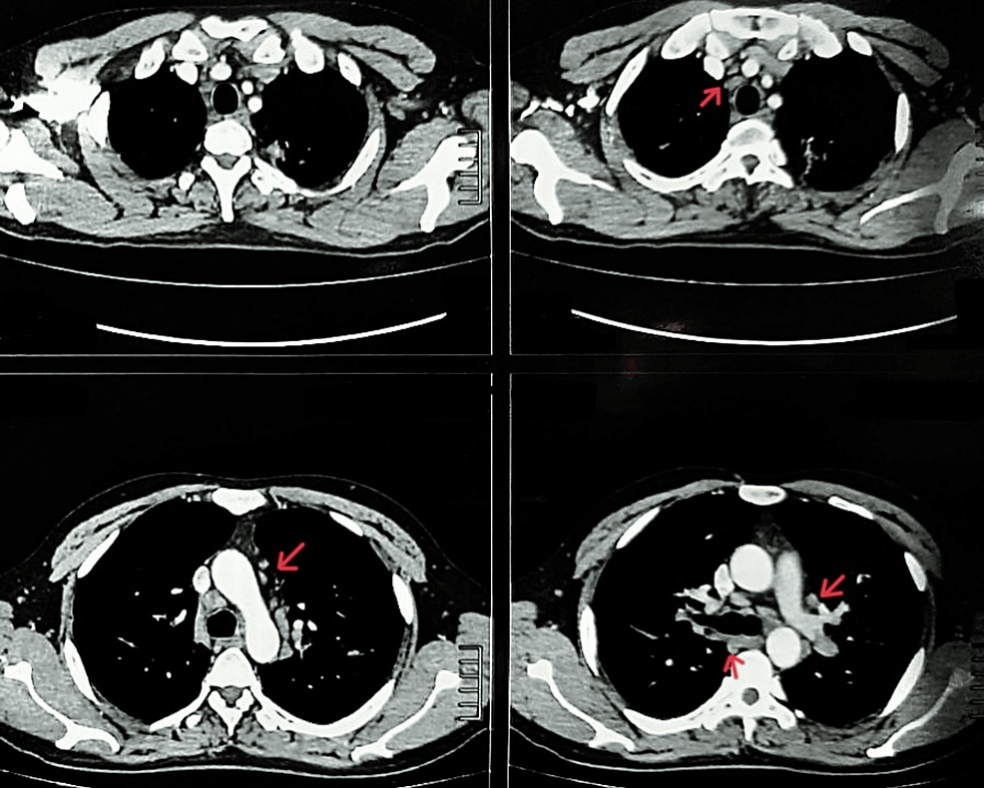
Computed tomography of the chest showing enlarged bilateral hilar lymph nodes.

**Figure 3:**
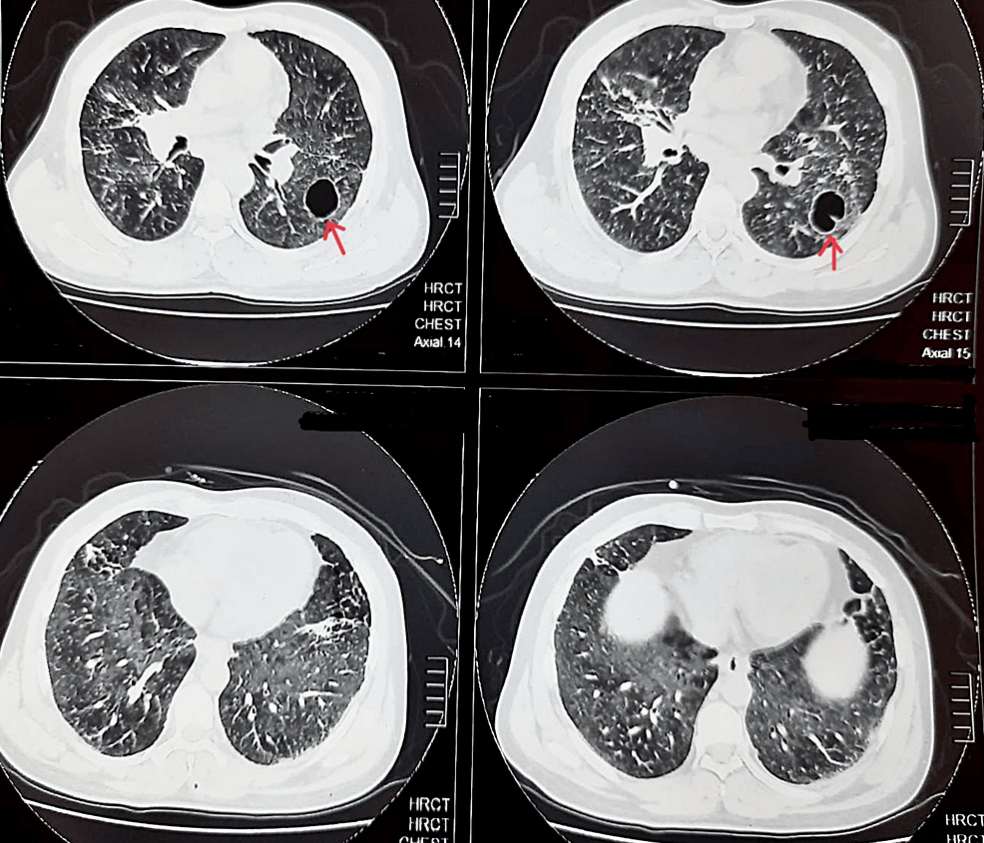
High-resolution computed tomography of the chest (axial view) showing cavitation, multiple pulmonary nodules, and interstitial thickening.

**Figure 4:**
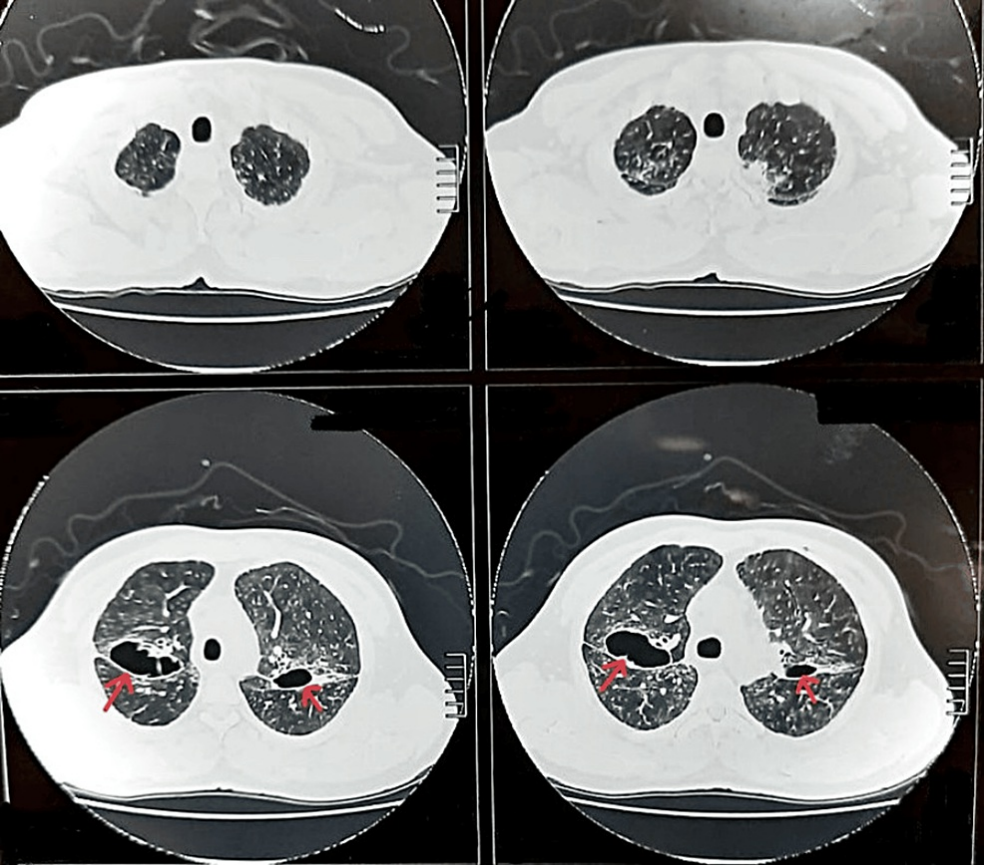
High-resolution computed tomography of the (axial view) showing bilateral cavitation, multiple pulmonary nodules, and interstitial thickening.

A high-resolution computed tomography (HRCT) of the chest revealed enlarged mediastinal and bilateral hilar lymph nodes (Figure 2). There was also the presence of bilateral perihilar consolidation along with cavitation in the upper lobes of both the right and left lung, with the diameter of left cavitation being 3 cm × 4 cm × 2 cm with a wall thickness of around 3 mm and the right one being 6 cm × 2 cm × 4 cm with a wall thickness of around 3.4 mm (Figures 3, 4).

**Figure 5:**
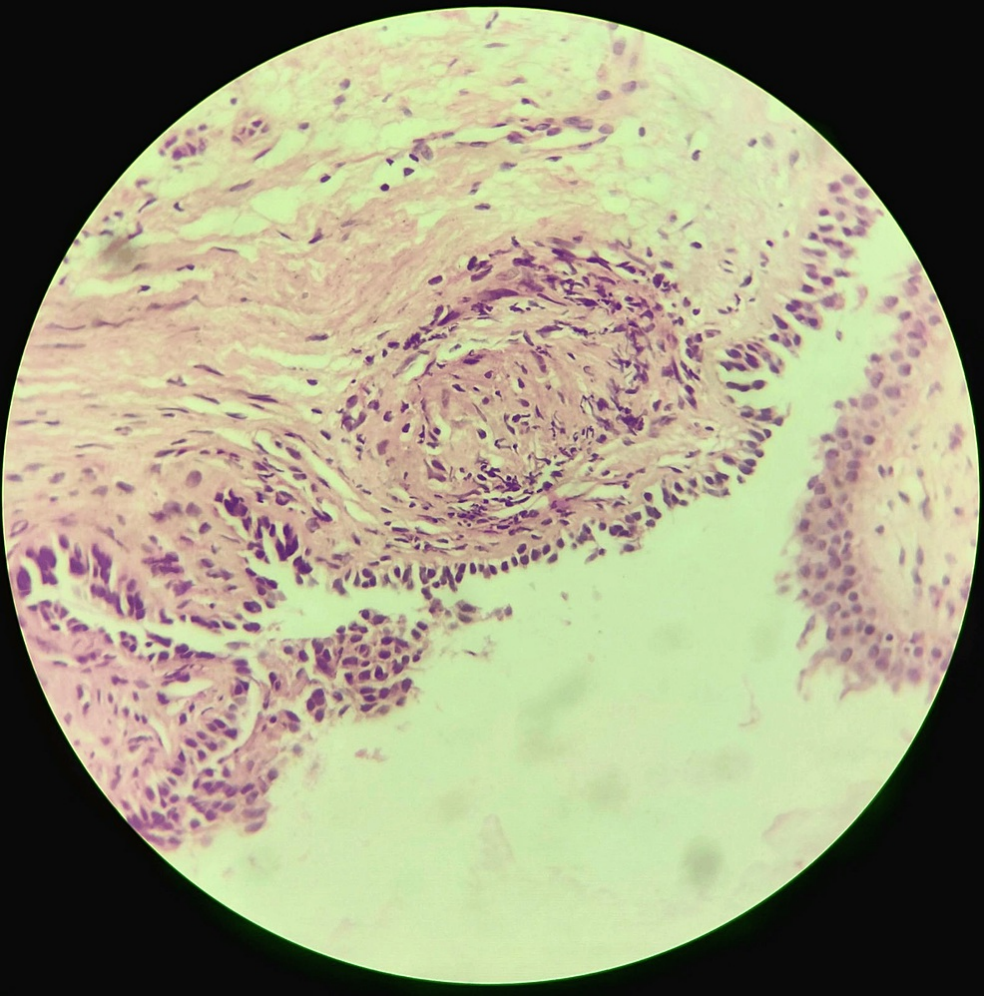
Histology from an endobronchial biopsy taken from the carina showing well-formed and non-necrotizing granulomas which were also embedded in the dense hyaline sclerosis (hematoxylin-eosin, 100×).

Flexible bronchoscopy with bronchoalveolar lavage (BAL) was obtained from the right upper lobe and left upper lobe of the lung and an endobronchial biopsy (EBB) from the carina was done. There was a nodular lesion on the posterior aspect of the left vocal cord and a tiny pustular mucosal lesion on the left main bronchus. The BAL for GeneXpert MTB/RIF, acid-fast bacilli, gram stain, and malignant cells were negative. There was no growth on routine sputum and fungal culture. EBB showed well-formed and non-necrotizing granulomas which were also embedded in the dense hyaline sclerosis (Figure 5).

His history, laboratory workup, radiological imaging, and biopsy findings were consistent with sarcoidosis. The patient was initiated on tablet prednisolone 40 mg on a tapering dosage for three months which led to significant improvement of his chief complaints.

## Discussion

Sarcoidosis is a granulomatous disease that can affect any organ system in the body and is without a known etiology [5]. Cavitary lesion as the primary presentation in sarcoidosis is rare and has been observed in fewer than 0.5% of people suffering from sarcoidosis [2]. In PCS, the granulomas in the lungs undergo central necrosis, resulting in the formation of cavities or holes within the lung tissue [4]. These cavities are similar to those seen in other conditions such as tuberculosis, lung abscesses, malignancy, and vasculitis. However, unlike tuberculosis or abscesses, the cavities in PCS do not contain infection. Thus, an extensive study such as BAL sample for GeneXpert MTB/RIF, acid-fast bacilli, gram stain, and culture and malignant cells to rule out bacterial, mycobacterial, and fungal infection and malignancy is needed [3]. Various hypotheses have been postulated to explain the occurrence of these types of cavities. These include bacterial infections, fungal infections, mycobacterium infections, and bullae formation which can be seen in chronic obstructive airway disease or because of traction secondary to fibrosis and development of cystic bronchiectasis [6].

Pulmonary sarcoidosis classically presents with constitutional symptoms such as a dry or persistent cough, shortness of breath which worsens with activity, chest pain, and wheezing. There can be additional symptoms such as painful red bumps on different parts of the body (erythema nodosum), irritation of the eyes, weight loss, and abdominal pain. As mentioned, sarcoidosis can also affect other systems of the body causing renal stones, joint pain and stiffness, an increase in calcium levels in the body, and heart failure [5].

The diagnosis of sarcoidosis can be made by examining the clinical signs and symptoms, radiological changes, and histopathological findings, as well as by excluding other diseases that may present with similar symptoms [5]. The laboratory assay should include a total blood count, a kidney function test, a liver function test, serum and urinary calcium levels, vitamin D levels, and ACE levels [7]. Similarly, ACE levels have a sensitivity of 60% and a specificity of 70%. Routine blood findings may show hypercalcemia and a raised erythrocyte sedimentation rate [2].

HRCT of the chest, bronchoscopy with EBB along with BAL, and pulmonary function test (PFT) should also be carried out to rule out other diseases.

Pulmonary sarcoidosis is divided into four stages according to the traditional Scadding staging system which is based on chest radiographic findings. Stage I includes normal chest radiograph findings, stage II includes bilateral hilar lymphadenopathy, stage III includes pulmonary infiltrates alone, and stage IV involves fibrosis as well. However, PCS does not fit into the traditional Scadding staging system. Instead, it is considered an unusual manifestation of the disease.

Stages I and II resolve on their own. The indications for medical treatment include a decrease in quality of life due to cough or dyspnea; ocular, liver, neurological, or cardiac involvement; calcium dysregulation; fatigue; and small fiber neuropathy [8]. No acceptance has been seen of the treatment algorithm, the total duration, and the dosage of drugs used for the treatment of sarcoidosis. Corticosteroids are regarded as the drug of choice. In individuals with pulmonary sarcoidosis, including PCS, more than 70% usually respond to corticosteroid therapy. Furthermore, it has been seen that there might be a 25-50% chance of recurrence if the corticosteroid dose is reduced or discontinued altogether [9]. The dosage of corticosteroid used is 20-40 mg/day which is tapered if the condition of the individual has improved [10]. Treatment response can be observed by examining the clinical response, PFT, and CXR every three weeks. The accepted corticosteroid duration for treatment and dosage reduction protocol differs greatly. Winterbauer et al. suggest that the treatment should be continued for one year due to the risk of relapse, and Huninghake et al. suggest that the dosage reduction and discontinuation should be done within six months of starting treatment [11,12]. Other second-line drugs are also available that can be considered in individuals with the persistence of symptoms while on corticosteroids, uncontrolled disease, exacerbation after dosage reduction, and side effects due to corticosteroids. These second-line drugs include hydroxychloroquine, methotrexate, chloroquine, cyclosporine, azathioprine, pentoxifylline, and minocycline [13].

The severity of pulmonary cavitary sarcoidosis can range from accidental findings while doing a CXR in asymptomatic individuals to a chronic progressive disease that can be resistant to treatment. Pulmonary fibrosis, pulmonary arterial hypertension, and disease processes extending to both the lungs predict poor clinical outcomes in patients. Cavitation may eventually lead to secondary infections such as aspergilloma, hemoptysis, and pneumothorax. Similarly, respiratory failure is the leading cause of death due to sarcoidosis.

## Conclusions

An atypical presentation of pulmonary sarcoidosis such as cavitary lesions can be difficult to distinguish because of the similarities in the clinical and radiographical features with other cavitary lesions of the lung. For this reason, with this case report, we would like to highlight the significance of acknowledging pulmonary sarcoidosis as a differential of cavitary lung disease. Furthermore, as the understanding of this rare presentation grows, further research and collaboration are necessary to unravel the pathogenesis and optical management strategies for this unique subset of sarcoidosis.
